# Meta-Local Density Functionals: A New Rung on Jacob’s Ladder

**DOI:** 10.1021/acs.jctc.0c01147

**Published:** 2021-01-27

**Authors:** Susi Lehtola, Miguel A. L. Marques

**Affiliations:** † Department of Chemistry, University of Helsinki, P.O. Box 55 (A.I. Virtasen aukio 1), FI-00014 University of Helsinki, Finland; ‡ Institut für Physik, Martin-Luther-Universität Halle-Wittenberg, 06120 Halle, Saale, Germany

## Abstract

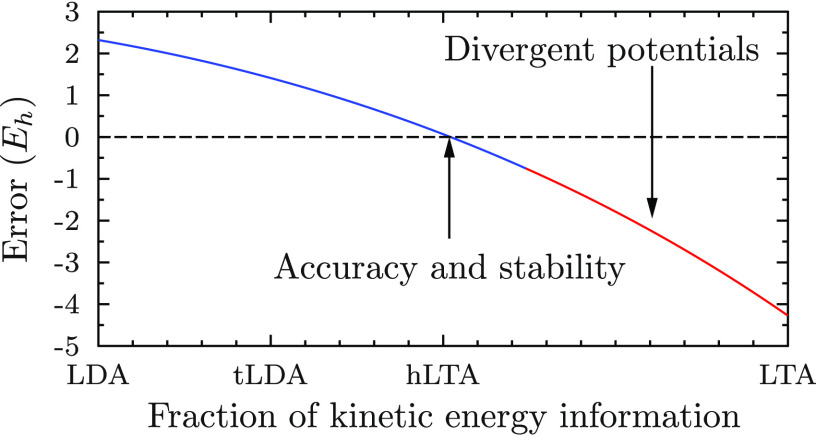

The homogeneous electron gas (HEG) is a key ingredient in the construction of most exchange-correlation functionals of density-functional theory. Often, the energy of the HEG is parameterized as a function of its spin density *n*_σ_, leading to the local density approximation (LDA) for inhomogeneous systems. However, the connection between the electron density and kinetic energy density of the HEG can be used to generalize the LDA by evaluating it on a geometric average *n*_σ_^avg^(**r**) = *n*_σ_^1–*x*^(**r**)*ñ*_σ_^*x*^(**r**) of the local spin density *n*_σ_(**r**) and the spin density *ñ*_σ_(**r**) of a HEG that has the local kinetic energy density τ_σ_(**r**) of the inhomogeneous system. This leads to a new family of functionals that we term meta-local density approximations (meta-LDAs), which are still exact for the HEG, which are derived only from properties of the HEG and which form a new rung of Jacob’s ladder of density functionals [AIP Conf. Proc.2001, 577, 1]. The first functional of this ladder, the local τ approximation (LTA) of Ernzerhof and Scuseria [J. Chem. Phys.1999, 111, 911] that corresponds to *x* = 1 is unfortunately not stable enough to be used in self-consistent field calculations because it leads to divergent potentials, as we show in this work. However, a geometric averaging of the LDA and LTA densities with smaller values of *x* not only leads to numerical stability of the resulting functional but also yields more accurate exchange energies in atomic calculations than the LDA, the LTA, or the tLDA functional (*x* = 1/4) of Eich and Hellgren [J. Chem. Phys.2014, 141, 22410725494732]. We choose *x* = 0.50, as it gives the best total energy in self-consistent exchange-only calculations for the argon atom. Atomization energy benchmarks confirm that the choice *x* = 0.50 also yields improved energetics in combination with correlation functionals in molecules, almost eliminating the well-known overbinding of the LDA and reducing its error by two thirds.

## 1 Introduction

The homogeneous electron gas (HEG) has a special place in the history of the study of many-electron systems in general, and density-functional theory in particular.^1,2^ In fact, the development of accurate exchange-correlation functionals typically begins with the local (spin) density approximation (LDA), whose construction is based on the exchange-correlation energy of the HEG. This is then modified by an enhancement factor that depends on the gradient of the density in the generalized gradient approximation (GGA); the mega-GGA approximation adds further dependence on the local kinetic energy density and/or the Laplacian of the electron density.^3−5^

LDAs, GGAs, and meta-GGAs form the first three rungs of the so-called Jacob’s ladder of the density-functional theory,^6^ each rung generally leading to approximations with better accuracy. Although GGAs and meta-GGAs add more physical information into the density-functional approximation (DFA), they are typically constructed to maintain the exactness for the exchange-correlation energy of the HEG. In fact, it can be even argued that this is one of the most important exact conditions that a functional should fulfill.

In this work, we investigate the accuracy of an ansatz, which, like the LDA, is derived from considerations of the HEG only, but which adds a further dependence on the local kinetic energy density as in meta-GGAs. These functionals, which we term meta-LDA functionals, thus constitute a new rung on Jacob’s ladder of functionals, which is shown to have an accuracy between those of LDAs and GGAs.

The work is organized as follows. We will describe the theory behind the meta-LDA approach in Section 2. The implementation of the meta-LDA functionals and the details of our computations are described in Section 3. The accuracy of the novel functionals is then assessed by benchmarking the exchange energies of atoms and atomization energies of molecules in Section 4. A brief summary and conclusions are presented in Section 5. Atomic units are used throughout the manuscript, unless specified otherwise.

## 2 Theory

The LDA for the exchange energy is derived for the HEG as^7,8^
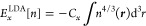
1where
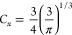
2The kinetic energy density of the gas is also known
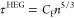
3where
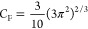
4Since eq 3 establishes a link between the kinetic energy density and the electron density, Ernzerhof and Scuseria^9^ proposed an exchange functional similar to eq 1, where eq 3 is used to replace the local density dependence by
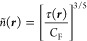
5yielding the local τ approximation (LTA) exchange functional
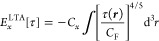
6

On the basis of the work of Ernzerhof and Scuseria, Eich and Hellgren^10^ suggested another exchange functional, where only the energy per unit particle is written as a function of the fictitious density of eq 5, yielding the tLDA exchange functional
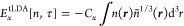
7

In this work, we show the power of this idea by generalizing the approach of Ernzerhof, Scuseria, Eich, and Hellgren. We thus replace the electron density by an effective density *n*^eff^(**r**) formed as a weighted combination of the electron density *n*(***r***) and the fictitious density computed from τ(***r***) as
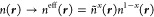
8This form interpolates between the LDA (*x* = 0), tLDA (*x* = 1/4), and LTA (*x* = 1) in the case of the exchange functional. Furthermore, it can also be employed within any LDA correlation functional, allowing us to generate a complete exchange-correlation ansatz.

We note here that the family of functionals generated by eq 8 is actually a member of a general family of functionals that have the form of an LDA, but which are based on a transformed density variable
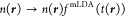
9where *t*(***r***) is the (dimensionless) reduced kinetic energy density
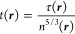
10It is easily seen that the LDA functionals operating on a density transformed according to eq 9 are exact for the HEG if the function *f*^mLDA^ reduces to one for the HEG, i.e.,
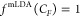
11Because this procedure generates a meta-GGA-type functional without gradient dependence from a LDA, we will term these functionals as meta-LDAs.

## 3 Computational Details

The effective density of eq 8 can be rewritten in the form of eq 9 as
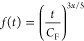
12The resulting meta-LDA version of the local exchange functional can be easily rewritten in terms of an enhancement function
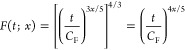
13The generalization of the Perdew–Wang 1992 correlation functional^11^ is equally trivial; the density used to evaluate the energy density is merely re-expressed using eq 8. These new functionals have been implemented in version 5.1.0 of the Libxc library of exchange-correlation functionals.^12^ In Libxc, the derivatives of the functional are evaluated analytically using the Maple symbolic algebra program, as is the case for all other functionals in Libxc as well. Combined with a basis set, these derivatives can be used to minimize the total energy variationally with respect to the orbital coefficients within a self-consistent field approach; we refer to ref (13) for discussion.

Fully numerical,^14^ fully variational calculations on closed and partially closed shell atoms from H to Sr were performed with the finite-element method as implemented in the HelFEM program,^15^ which allows for an efficient approach to the complete basis set limit.^16,17^ The atomic calculations employed five radial elements, yielding 69 numerical radial basis functions, which suffice to converge the energy to better than μ*E*_h_ precision for these systems.

Molecular calculations on the 183 non-multireference molecules in the W4-17 data set^18^ were performed with the Psi4 program.^19^ The Psi4 calculations employed the quadruple-ζ aug-pcseg-3 basis set^20−22^ and a (100, 590) quadrature grid. Density fitting^23^ was used to accelerate the Psi4 calculations; a universal auxiliary basis set was used for this purpose.^24^

## 4 Results

### 4.1 Atomic Calculations

**Figure 1:**
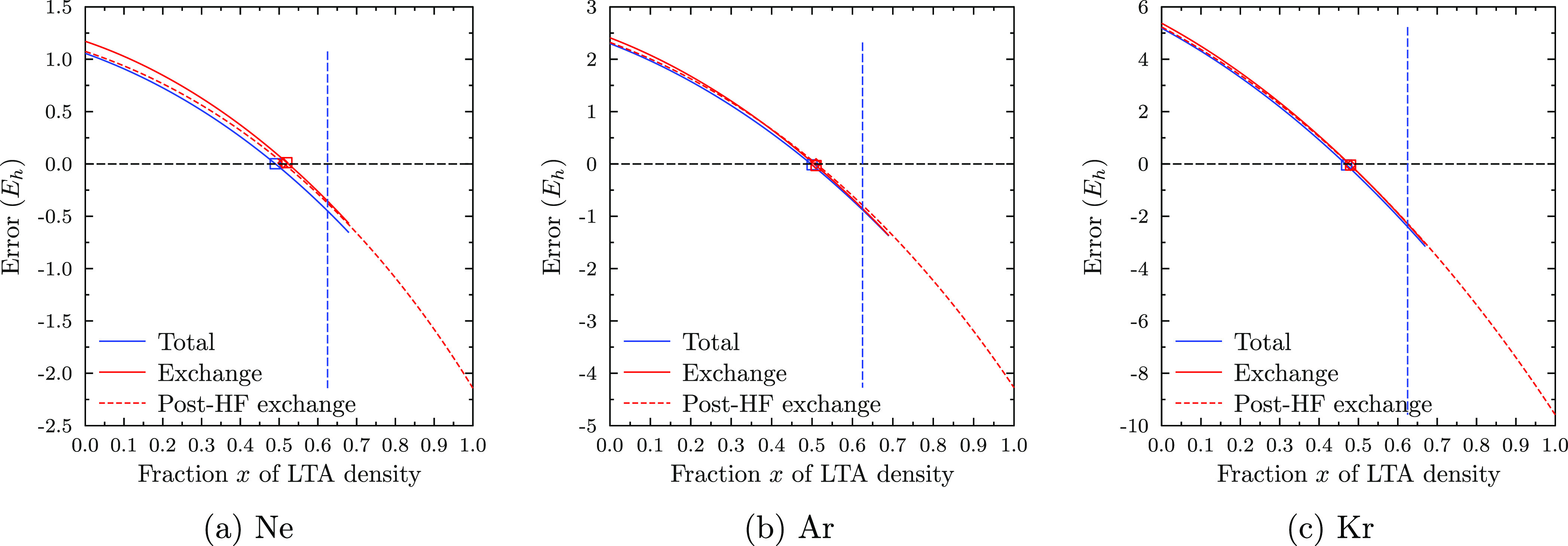
Errors in the self-consistent total (blue solid line) and exchange (red solid line) energies of Ne, Ar, and Kr, as well as in the perturbative exchange energy calculated on top of the HF density (dashed red line). The vertical dashed blue line shows the critical value *x* = 0.625, see the main text. The location of the smallest error for the self-consistent total and exchange energies are shown as blue and red squares, respectively, and that for the perturbative exchange energy as red diamonds; however, since the optimal value is close to *x* = 1/2 for all cases, the markers are on top of each other.

The errors of exchange-only density-functional calculations compared to the unrestricted Hartree–Fock (HF) total and exchange energies for atoms from H to Sr were studied with HelFEM; the reference unrestricted HF total energies have been recently reported in ref (17). Due to the similarity of the results, data is shown here only for the noble gases Ne, Ar, and Kr in Figure 1; the rest of the data can be found in the Supporting Information. In addition to the self-consistent data, Figure 1 also shows the perturbative evaluation of the exchange energy computed on top of the HF density.

Following Becke^25^ and Sun et al.^26^ among others, we fit the parameter *x* for our meta-LDAs by optimizing the total energy of the argon atom to the Hartree–Fock reference value, leading to the choice *x* = 0.50. It is noteworthy that in addition to being quasi-optimal for all systems, *x* = 0.50 is also numerically stable for all of the studied atoms. Finally, it also leads to uniformly smaller errors in the exchange energy than in the LDA and tLDA, which uniformly underestimate the energy, while LTA grossly overestimates the energy.

As already implied above, the self-consistent calculations diverge for large fractions *x* of the LTA density. We have analyzed the instability observed in the calculations; see the Appendix for a formal analysis. It turns out that the functional form is inherently unstable for *x* > 0.625, since for such values of *x*, the potentials corresponding to both *n* and τ diverge asymptotically to −∞ for *r* → ∞. However, it is clear from the results that the optimal value of *x* for the exchange functional is found at *x* < 0.625.

### 4.2 Molecular Calculations

The application of the functional to atomization energies
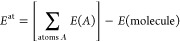
14of the non-multireference part of W4-17 yields the errors
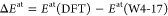
15shown in Table 1. Due to the higher cost of the molecular calculations compared to that of the atomic calculations, the new family of meta-LDA functionals is only studied at select points, which suffice for the present purposes of showing the proof of concept. The points at which the meta-LDAs are evaluated are indicated by a prefix to the name of the exchange and correlation functionals: data are presented for the LDA exchange functional as qLTA (same as Eich and Hellgren’s tLDA), tLTA, and hLTA, which stand for *x* = 1/4, *x* = 1/3, and *x* = 1/2, respectively. Data is given both for exchange-only calculations, and for combinations with the Perdew–Wang (PW92) correlation functional^11^ that also admits meta-LDA generalizations to qPW92, tPW92, and hPW92 for *x* = 1/4, *x* = 1/3, and *x* = 1/2, respectively.

**Table 1:** Mean Absolute Error (MAE) and Mean Error (ME) in Atomization Energies of the Non-Multireference Part of the W4-17 Test Set, Computed in the aug-pcseg-3 Basis with Density Fitting and a (100, 590) grid^c^

(A) Results for Exchange-Only Calculations			
functional	*x*	MAE (kcal/mol)	ME (kcal/mol)
LDA exchange		28.966	–12.015
hLTA exchange^a^	1/2	71.235	–67.512
tLTA exchange	1/3	47.504	–35.863
qLTA exchange^b^	1/4	42.181	–26.070
HF		144.848	–144.848
B88 exchange		98.177	–98.177
PBE exchange		87.958	–87.958

(B) Results for Exchange-Correlation Functionals				
functional	*x*_exchange_	*x*_correlation_	MAE (kcal/mol)	ME (kcal/mol)
LDA-PW92			79.879	79.879
qLTA-qPW92	1/4	1/4	61.089	60.897
tLTA-tPW92	1/3	1/3	50.207	49.494
hLTA-PW92	1/2	0	31.388	23.913
hLTA-hPW92	1/2	1/2	26.907	14.088
B88-P86			19.173	18.899
PBE-PBE			18.028	17.052
TPSS-TPSS			12.427	11.180
B88-LYP			8.176	1.714

a The data for the exchange-only hLTA calculation excludes CH_2_NH_2_ for which the SCF procedure did not converge.

b qLTA is the same as the tLDA of Eich and Hellgren.

c The data is divided into exchange-only calculations (A), and calculations including both exchange and correlation (B). See the main text for the legend of the functionals shown. To clarify the notation, the used values for *x* in the meta-LDA exchange and correlation functionals are also shown.

For comparison, data is also included for the Perdew–Burke–Ernzerhof exchange-correlation functional;^27,28^ combinations of the Becke’88 (B88) exchange functional,^25^ with the Perdew’86^29,30^ (P86) and Lee–Yang–Parr^31^ (LYP) correlation functionals; as well as the Tao–Perdew–Staroverov–Scuseria (TPSS) exchange-correlation functional.^32,33^

Starting out with the basics, the table demonstrates the well-known characteristics of HF and LDA: HF severely underbinds molecules due to the complete neglect of electronic correlation effects, while LDA overbinds them. Due to the overbinding, exchange-only LDA calculations are more accurate than those that explicitly include also correlation contributions, although the LDA exchange by itself is slightly underbinding. In contrast, while gradient-corrected exchange functionals yield bad results if used alone, when they are combined with a good gradient-corrected correlation functional, they achieve good accuracy. Jacob’s ladder^6^ is also visible in the results: more accurate atomization energies are obtained in the sequence LDA → PBE → TPSS.

**Figure 2:**
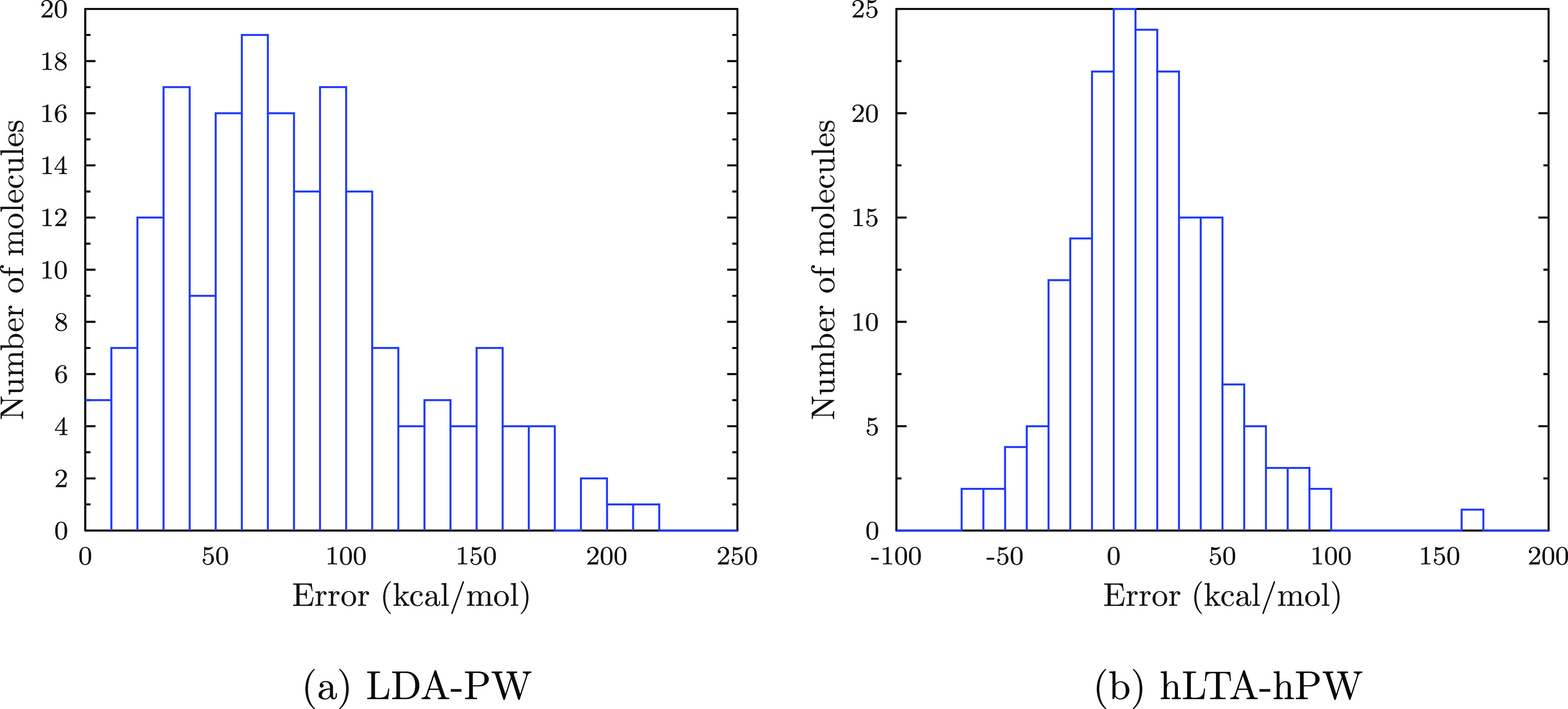
Error histograms for the atomization energies of the non-multireference part of W4-17 in the aug-pcseg-3 basis set.

Interestingly, also the meta-LDA functionals show monotonic behavior. Going from LDA to qLTA to tLTA and, finally, hLTA in exchange-only calculations leads to systematically increasing underbinding. The same effect holds also in the presence of correlation: while LDA-PW92 is greatly overbinding, as already established above, the overbinding decreases systematically in the sequence LDA-PW92 → qLTA-qPW92 → tLTA-tPW92 → hLTA-hPW92. Like in the case of the atomic exchange energies, the half-and-half *x* = 1/2 mixture of the electron density with the τ-based density as in the hLTA-hPW92 functional yields the best results with a mean absolute error almost 3 times smaller than that in the original LDA-PW92 calculation. This finding is underlined by the error histograms shown in Figure 2: while LDA-PW is consistently overbinding, the errors for hLTA-hPW are almost symmetric, even though the error scale is still large compared to that of the established GGA functionals.

## 5 Summary and Conclusions

We have proposed a new class of functionals as generalizations of the established class of local density approximations (LDAs) by including a fraction *x* of fictitious density computed from the local kinetic energy density via a relation derived for the homogeneous electron gas (HEG). The resulting so-called meta-LDA functionals maintain the exactness of LDA for the HEG, and are derived from HEG data only (with the exception of the one parameter *x* that is fitted to the total exchange-only energy of the argon atom) but afford much improved accuracy for inhomogeneous systems, thus forming a new rung on Jacob’s ladder of density functionals in between LDAs and GGAs. Benchmarks on both perturbative and self-consistent atomic exchange energies and molecular atomization energies in the presence of a correlation functional showed that the half-and-half ratio *x* = 1/2 yields quasi-optimal results for both atoms and molecules, almost fully eliminating the overbinding of LDA and reducing the mean absolute error in the atomization energies to a third of the original.

Meta-LDAs could also be seen as a better starting point for the inclusion of an extra dependency in the gradient of the density (as in a standard GGA), and in the Laplacian of the density and the kinetic energy density (as in a standard meta-GGA). Due to the extra flexibility, we can expect that these perform better than the parent functionals. For example, the new degree of freedom introduced with the meta-LDAs could play an important role for, e.g., semi-empirical functionals fitted to the experimental data. In many of these cases (see for example in refs (34−36)), the functionals do not reduce to the LDA for homogeneous densities, as this would compromise the accuracy of the functional for other systems. By replacing the standard LDA with a meta-LDA form in full or in part could, in principle, obey the exact condition without compromising the accuracy, and at the same time increase the transferability of the functionals to solids. Of course, the GGA or meta-GGA enhancement functionals have to be redesigned (or at least reoptimized) to take the new form into account. Work along these lines has already started.

## Appendix: Instability of the Local τ Approximation

The Kohn–Sham electron density is known to behave asymptotically as  due to the highest occupied molecular orbital (HOMO), which behaves as .^37^ For simplicity, we will study hydrogenic orbitals of the form

16

to show that the exponentially decaying asymptotic region leads to problems for *r* → ∞ for the local τ exchange functional. The electron density of the wave function in eq 16 is

17

while the kinetic energy density is

18

The self-consistent implementation of the meta-LDAs is based on the potentials *v*_*n*_^σ^ and *v*_τ_^σ^, which are defined as the derivatives of the exchange energy density arising from the substitution of eq 8 into eq 1 with respect to *n*_σ_ and τ_σ_, respectively.^13^ It is easy to show using, e.g., computer algebra (we used Maple 2020 to obtain these results), that when evaluated on an electron density and kinetic energy density of the form of eqs 17 and 18, both of the potentials *v*_*n*_^σ^ and *v*_τ_^σ^ contain a factor of the form exp[(16*x* – 10)*r*ζ/15], which diverges in the limit *r* → ∞ whenever *x* > *x*_crit_ with the critical value *x*_crit_ = 5/8 = 0.625.

Interestingly, also the choice of a HOMO with a Gaussian form ψ_σ_ ∝ exp(−ζ*r*^2^) leads to divergent potentials, only now of a stronger kind exp[(16*x* – 10)*r*^2^ζ/15], and yields the same critical value *x*_crit_ = 5/8. In fact, it can be shown that *all* asymptotic wave functions of the kind ψ_σ_ ∝ exp(−ζ*r*^*p*^) with *p* > 0 lead to divergences of the kind exp[(16*x* – 10)ζ*r*^*p*^/15] in *v*_*n*_^σ^ and *v*_τ_^σ^. The total exchange energy, however, is finite in each case.

For *x* > *x*_crit_, one then has *v*_*n*_^σ^ → −∞ and *v*_τ_^σ^ → −∞ for *r* → ∞ because the potentials are negative everywhere (as expected for an exchange functional). This divergence causes convergence problems. Assuming an orthonormal basis set {χ_μ_}, the potentials *v*_*n*_^σ^ and *v*_τ_^σ^ contribute to the Kohn–Sham–Fock matrix as^13^

19

The tentative physical interpretation of the divergent negative potentials is that displacing electron density toward *r* → ∞ would lead to a decrease in the energy. Now, if a Gaussian-type or Slater-type orbital basis set is employed, χ_μ_ and its gradient will decay asymptotically as exp(−α_μ_*r*^2^) or exp(−ζ_μ_*r*), respectively, where α_μ_ and ζ_μ_ are the Gaussian- and Slater-type exponents, with analogous expressions for ∇χ_ν_. Evaluating eq 19 then requires quadrature of an expression with an exponentially decaying part and an exponentially increasing part, which is numerically unstable, as the resulting value may be either small or large. The finite-element calculations with HelFEM, in turn, feature localized basis functions also at large values of *r*. This leads to exponentially increasing elements of the Kohn–Sham–Fock matrix, making the self-consistent field algorithm unstable.

In contrast, the potentials arising in the asymptotic region for *x* < 0.625 decay exponentially (like they do in the local density approximation), making self-consistent field calculations stable.

## Supporting Information Available

Further plots on the accuracy of the meta-LDA exchange functional with varying fractions *x* of LTA density for atoms from H to Sr, and the raw data for the atomization energies (PDF)

## Notes

The data that supports the findings of this study is available within the article and its supporting material.
