# Supplementary material for: Meta-Local Density Functionals: A New Rung on Jacob’s Ladder
Source: J Chem Theory Comput. 2021 Jan 27;17(2):943–8. doi: 10.1021/acs.jctc.0c01147 (PMC8023657; doi:10.1021/acs.jctc.0c01147)
Supplement: Supplementary file 1 — ct0c01147_si_001.pdf [file ct0c01147_si_001.pdf]

Supporting Information: Meta-local density functionals: a new rung on Jacob's ladder

Susi Lehtola*,† and Miguel A. L. Marques‡

†*Department of Chemistry, University of Helsinki, P.O. Box 55 (A. I. Virtasen aukio 1), FI-00014 University of Helsinki, Finland*

‡*Institut für Physik, Martin-Luther-Universität Halle-Wittenberg, 06120 Halle (Saale), Germany*

E-mail: susi.lehtola@alumni.helsinki.fi

The errors of exchange-only density functional calculations compared to unrestricted HF total and exchange energies for atoms from H to Sr are shown in figure S1 for closed-shell atoms (excluding Ne, Ar, and Kr that were presented in the main text), and in figures S2 and S3 for the partially closed-shell atoms. In addition to the self-consistent data, figures S1 to S3 also show a perturbative evaluation of the exchange energy computed on top of the HF density. The full list of atomization energies is attached here in plain text.

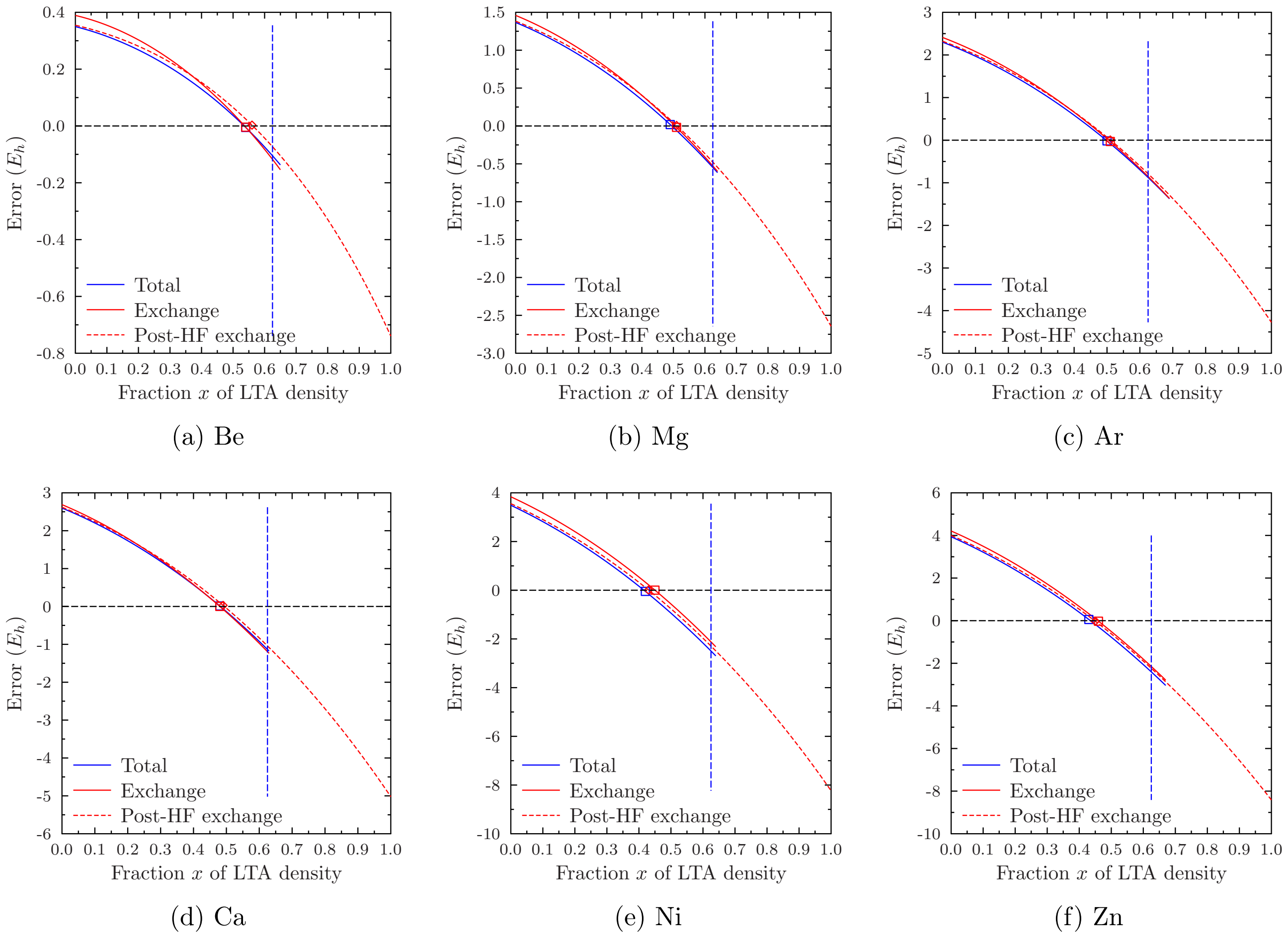

Figure S1: Errors in self-consistent total (blue solid line) and exchange (red solid line) energies of closed-shell atoms, as well as in the perturbative exchange energy calculated on top of the HF density (dashed red line). The location of the smallest error for the self-consistent total and exchange energies are shown as the blue and red squares, respectively, and the one for the perturbative exchange energy as red diamonds.

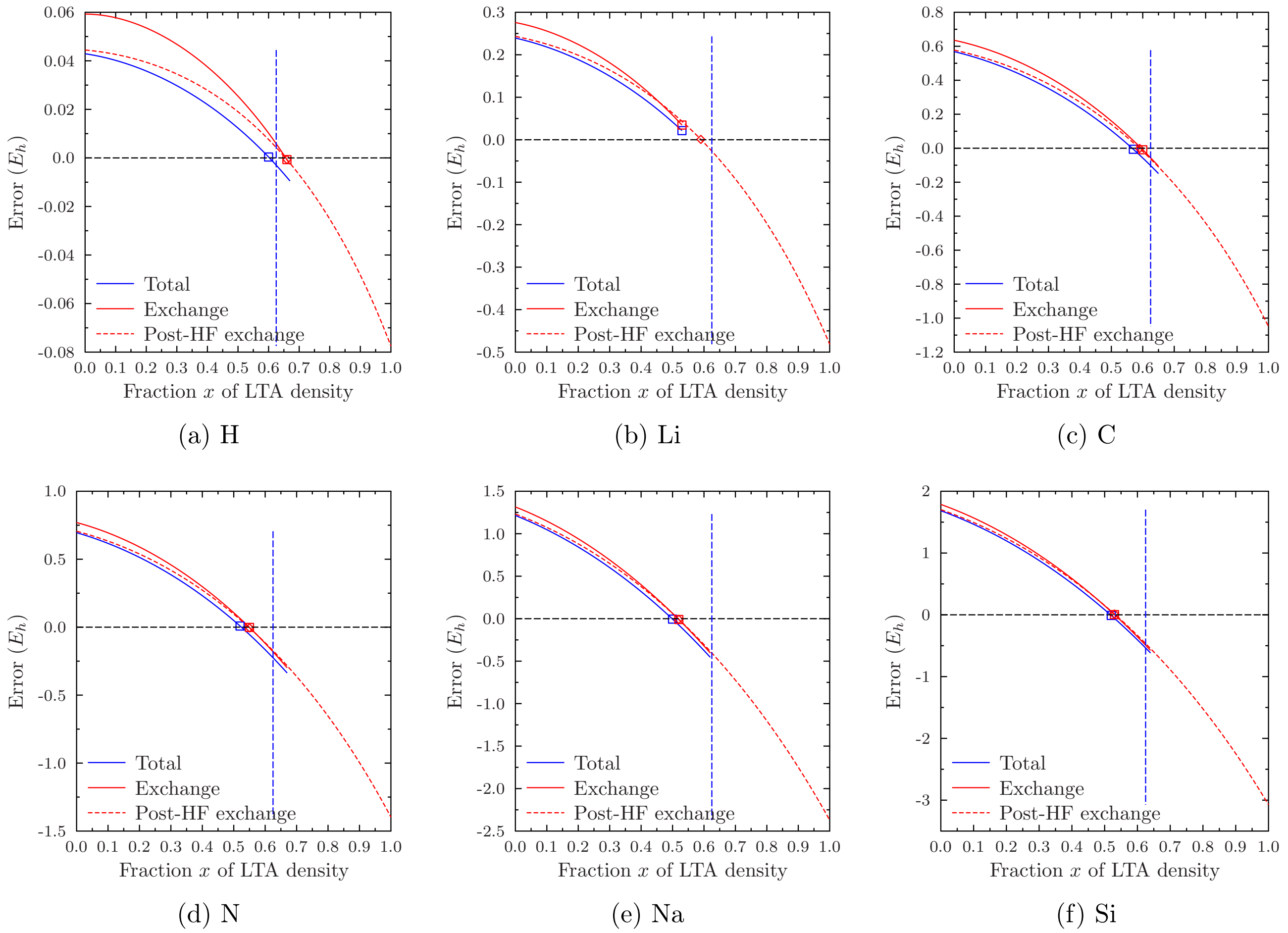

Figure S2: Errors in self-consistent total (blue solid line) and exchange (red solid line) energies of partially closed-shell atoms, as well as in the perturbative exchange energy calculated on top of the HF density (dashed red line). The location of the smallest error for the self-consistent total and exchange energies are shown as the blue and red squares, respectively, and the one for the perturbative exchange energy as red diamonds.

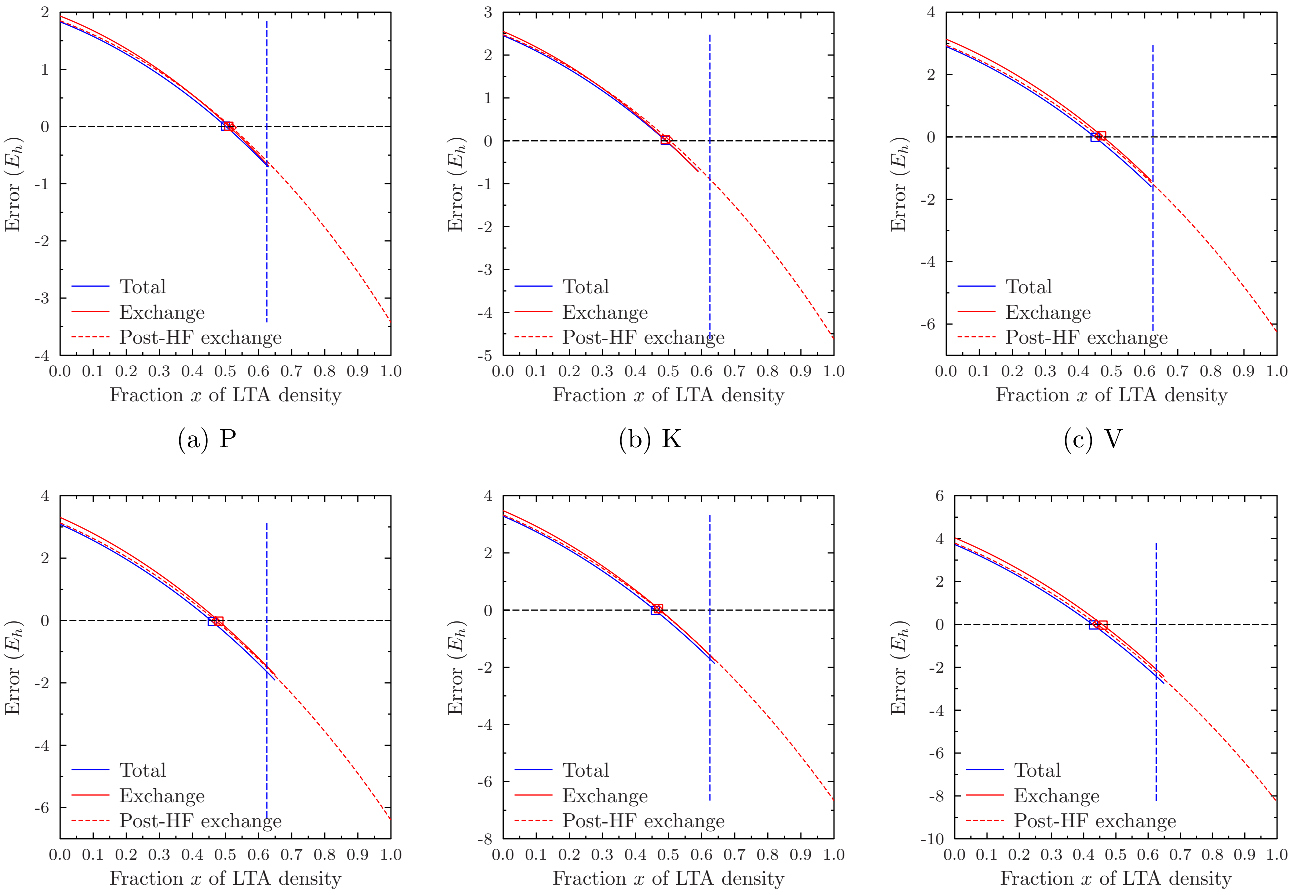

(a) P (b) K (c) V

(d) Cr (e) Mn (f) Cu

Figure S3: Errors in self-consistent total (blue solid line) and exchange (red solid line) energies of partially closed-shell atoms, as well as in the perturbative exchange energy calculated on top of the HF density (dashed red line). The location of the smallest error for the self-consistent total and exchange energies are shown as the blue and red squares, respectively, and the one for the perturbative exchange energy as red diamonds.
